# Supplementary material for: Signatures in domesticated beet genomes pointing at genes under selection in a sucrose-storing root crop
Source: BMC Biol. 2025 Oct 7;23:299. doi: 10.1186/s12915-025-02422-5 (PMC12505646; doi:10.1186/s12915-025-02422-5)
Supplement: Supplementary file 1 — Additional file 1: Table S1: Summary statistics of the paired-end Illumina raw reads without trimming from 290 accessions. Table S2: Summary statistics of the paired-end Illumina trimmed and cleaned reads from 290 accessions. Table S3: Summary statistics of the sample information, total mapped reads (%), sequencing depth and genome coverage (%) across 290 accessions. “NA” : Not Available. Table S4: Summary statistics of sequence variants (SNPs and INDELs) in 290 beet accessions compared to the EL10 reference genome. EL10.2_2 genome assembly comprises 568.8 Mb and is assembled into 18 pseudomolecules. In total, 564.2 Mb (99.2%) of the total genome assembly is organized into 9 main chromosomes (pseudomolecules), and the remaining 4.6 Mb of the genome is placed on 9 small unscaffolded pseudomolecules. Fig. S1: A bar plot illustrating the number of variants, including SNPs and INDELs, across all nine EL10 chromosomes in the Beta mini-core collection of 290 accessions. The Y-axis represents the total number of variants, and the X-axis corresponds to the nine EL10 chromosomes. Chromosome 9 exhibits the minimum number of variants, approximately 1.1 million, while Chromosome 5 displays the maximum number, around 1.5 million variants. On average, each chromosome harbors approximately 1.3 million variants. Table S5: Summary statistics of the number of SNPs and INDELs with their predicted effect using VEP across 290 accessions. Table S6: Summary statistics of the number of SNPs and INDELs within coding sequences with their predicted effect using VEP across 290 accessions. Fig. S2: Principal component analysis (PCA) of 290 beet accessions comparing second and third principal components. PC2 and PC3 represent the second and third components, accounting for 3.08% and 2.50% of the total variation, respectively. The colors represent different genetic clusters according to their beet type, with the following accessions: wild accession from the Atlantic (purple), Mediterranean (blue) [file 12915_2025_2422_MOESM1_ESM.zip › Beta_supplementary_figures_18.09.2025.pptx]

## Slide 1
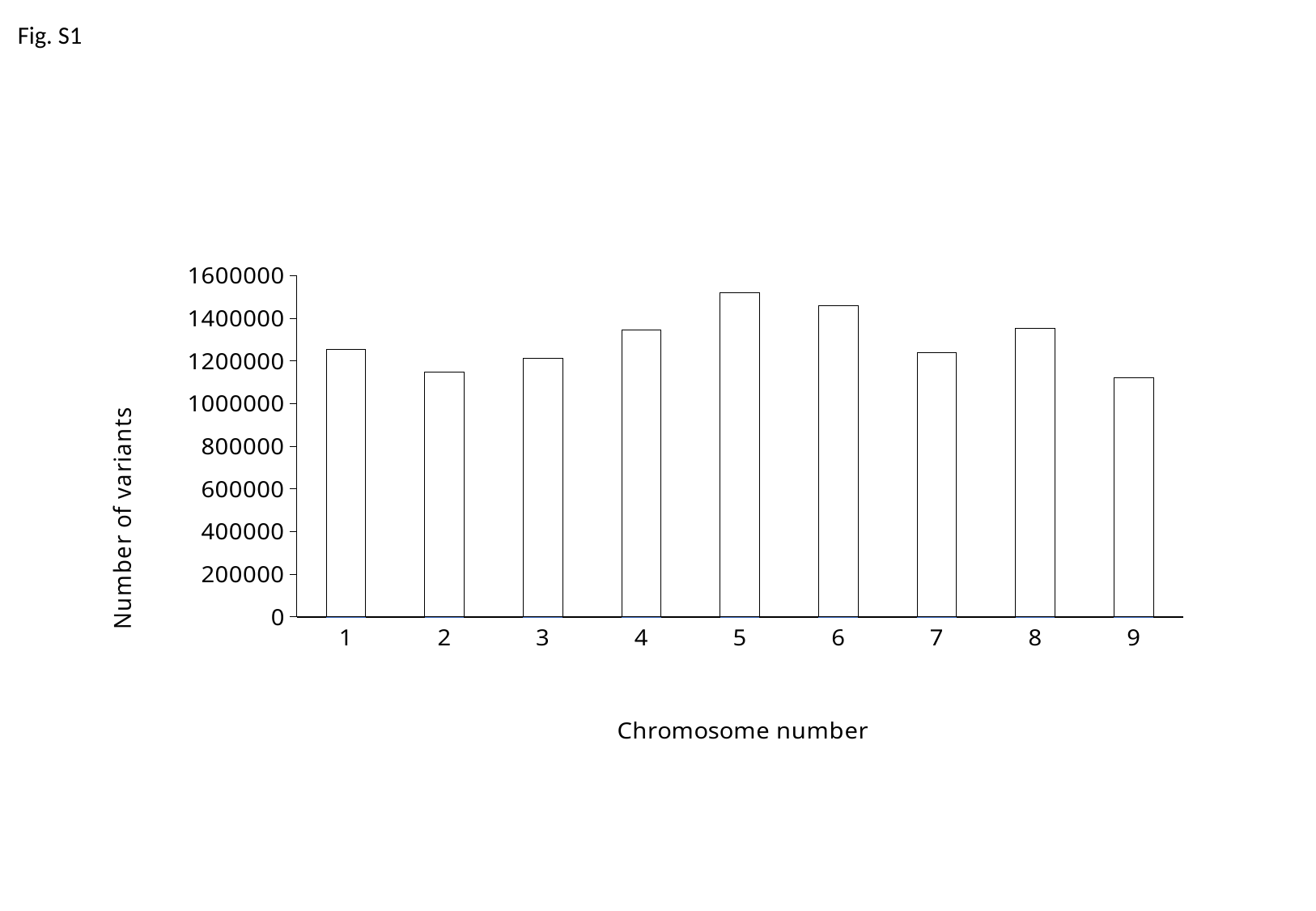

Fig. S1
### Chart
| Category | | |
|---|---|---|

## Slide 2
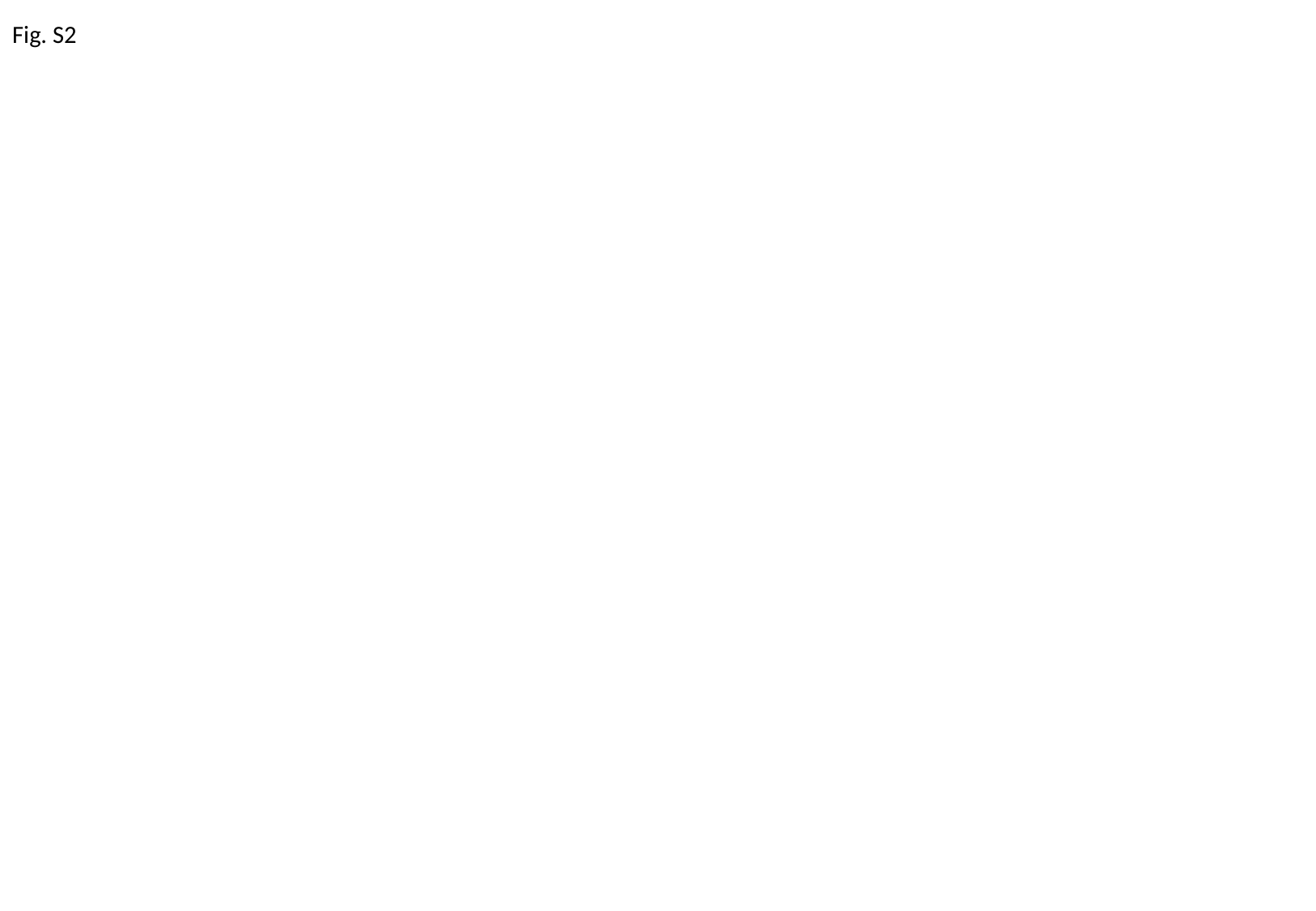

Fig. S2

## Slide 3
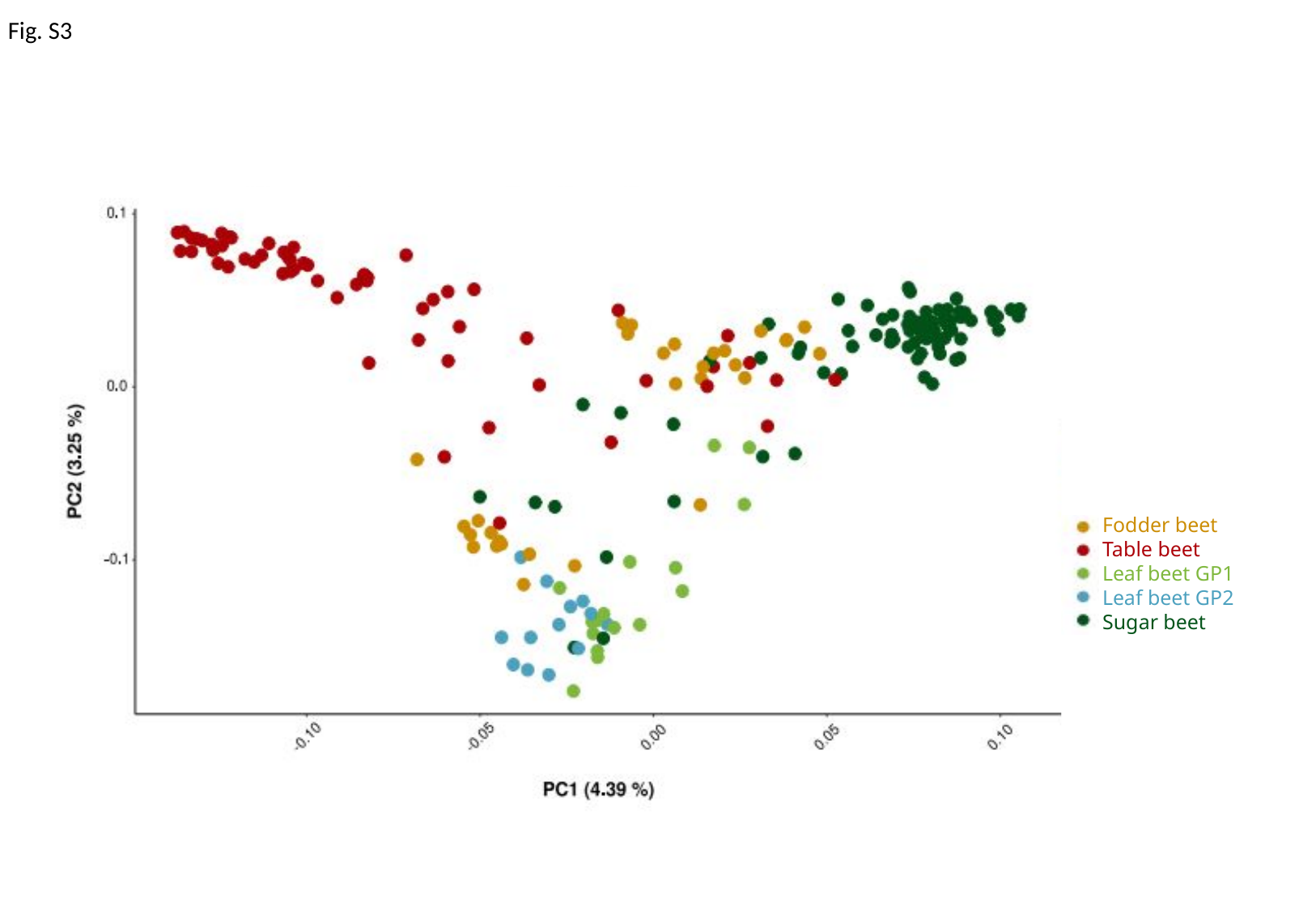

Fig. S3
Fodder beet
Table beet
Leaf beet GP1
Leaf beet GP2
Sugar beet

## Slide 4
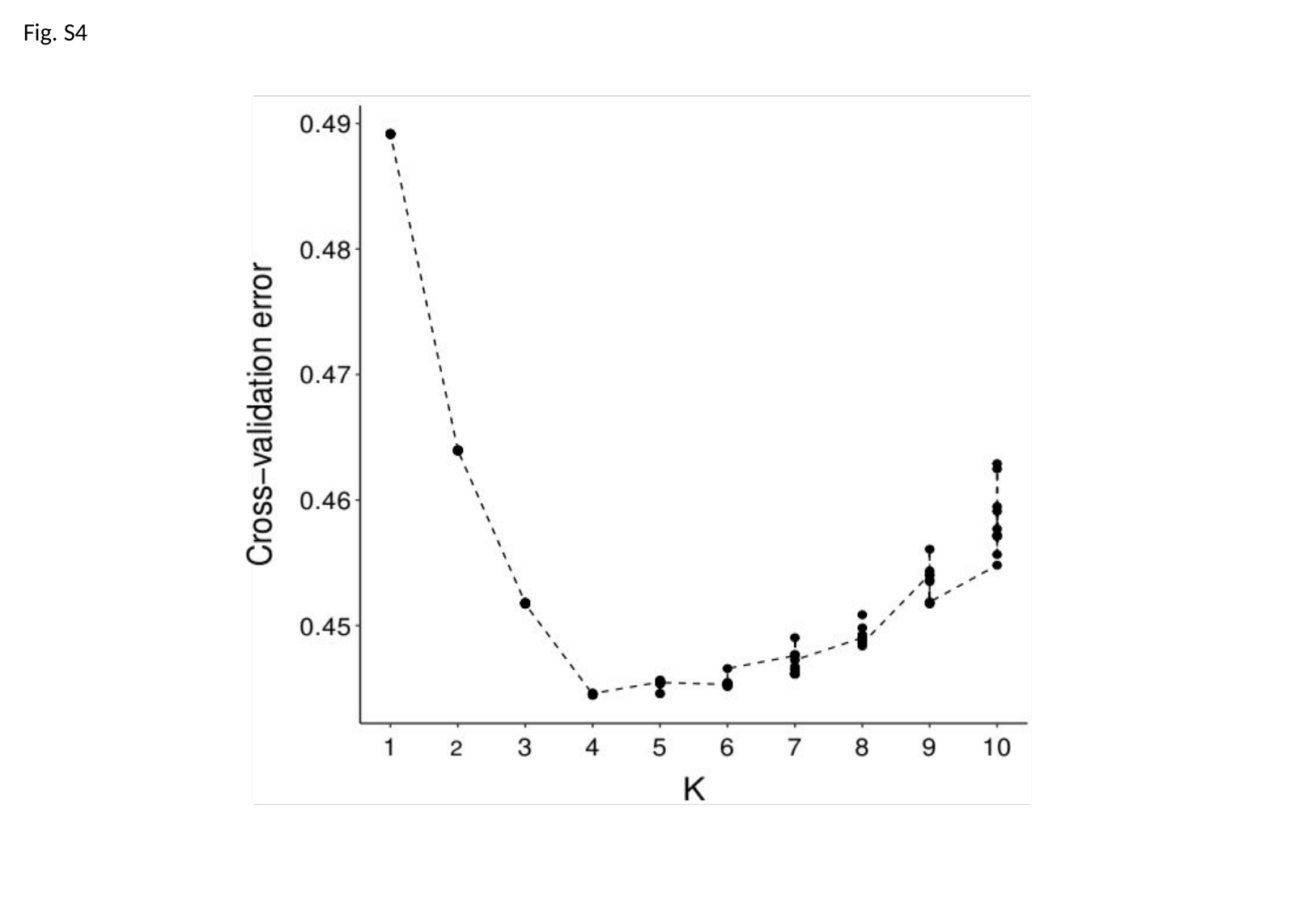

Fig. S4

## Slide 5
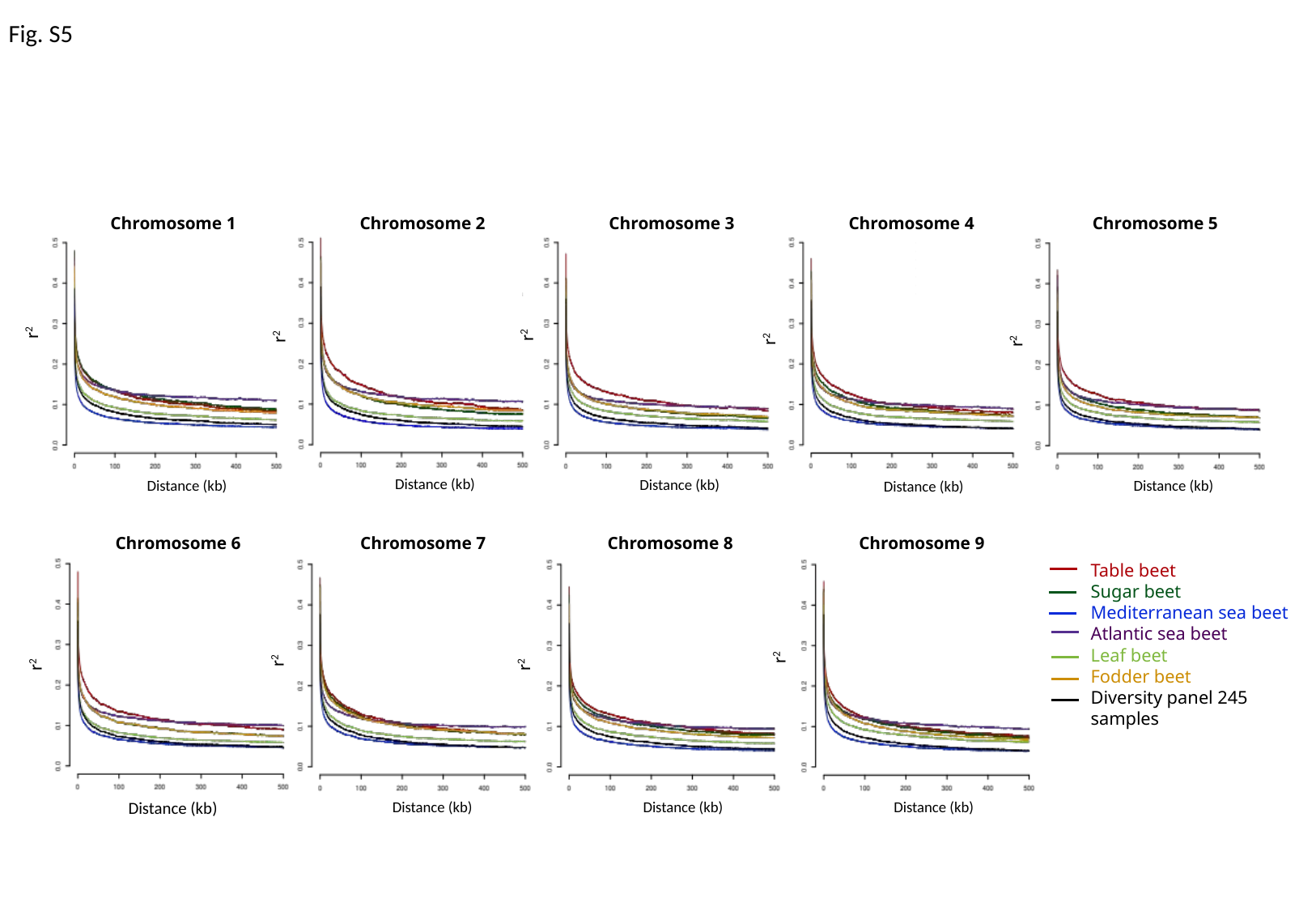

Fig. S5
Chromosome 4
Chromosome 5
Chromosome 2
Chromosome 3
Chromosome 1
r2
r2
r2
r2
r2
Distance (kb)
Distance (kb)
Distance (kb)
Distance (kb)
Distance (kb)
Distance (kb)
Chromosome 6
Chromosome 7
Chromosome 8
Chromosome 9
Table beet
Sugar beet
Mediterranean sea beet
Atlantic sea beet
Leaf beet
Fodder beet
Diversity panel 245 samples
r2
r2
r2
r2
Distance (kb)
Distance (kb)
Distance (kb)
Distance (kb)

## Slide 6
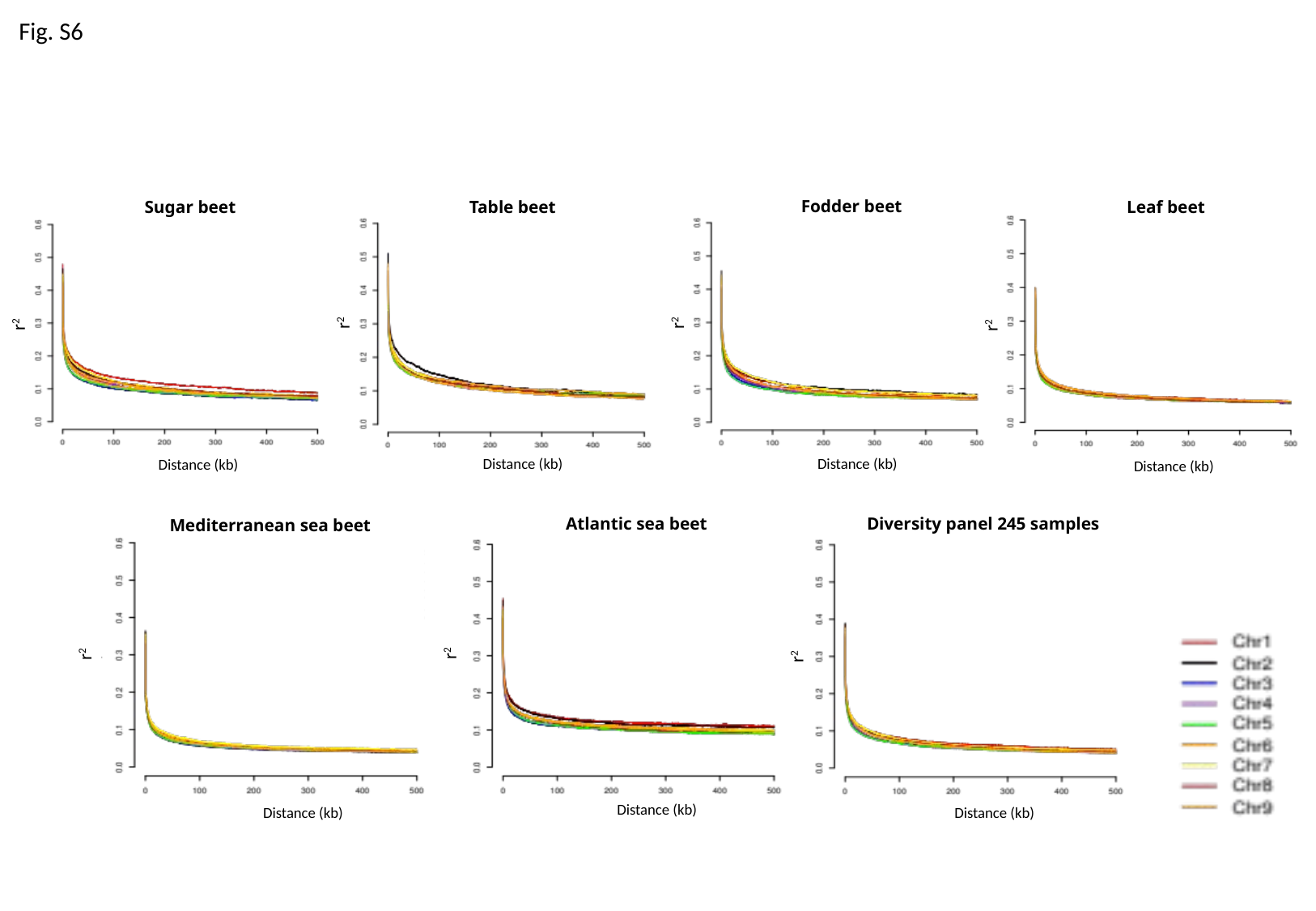

Fig. S6
Fodder beet
Leaf beet
Table beet
Sugar beet
r2
r2
r2
r2
Distance (kb)
Distance (kb)
Distance (kb)
Distance (kb)
Atlantic sea beet
Diversity panel 245 samples
Mediterranean sea beet
r2
r2
r2
Distance (kb)
Distance (kb)
Distance (kb)

## Slide 7
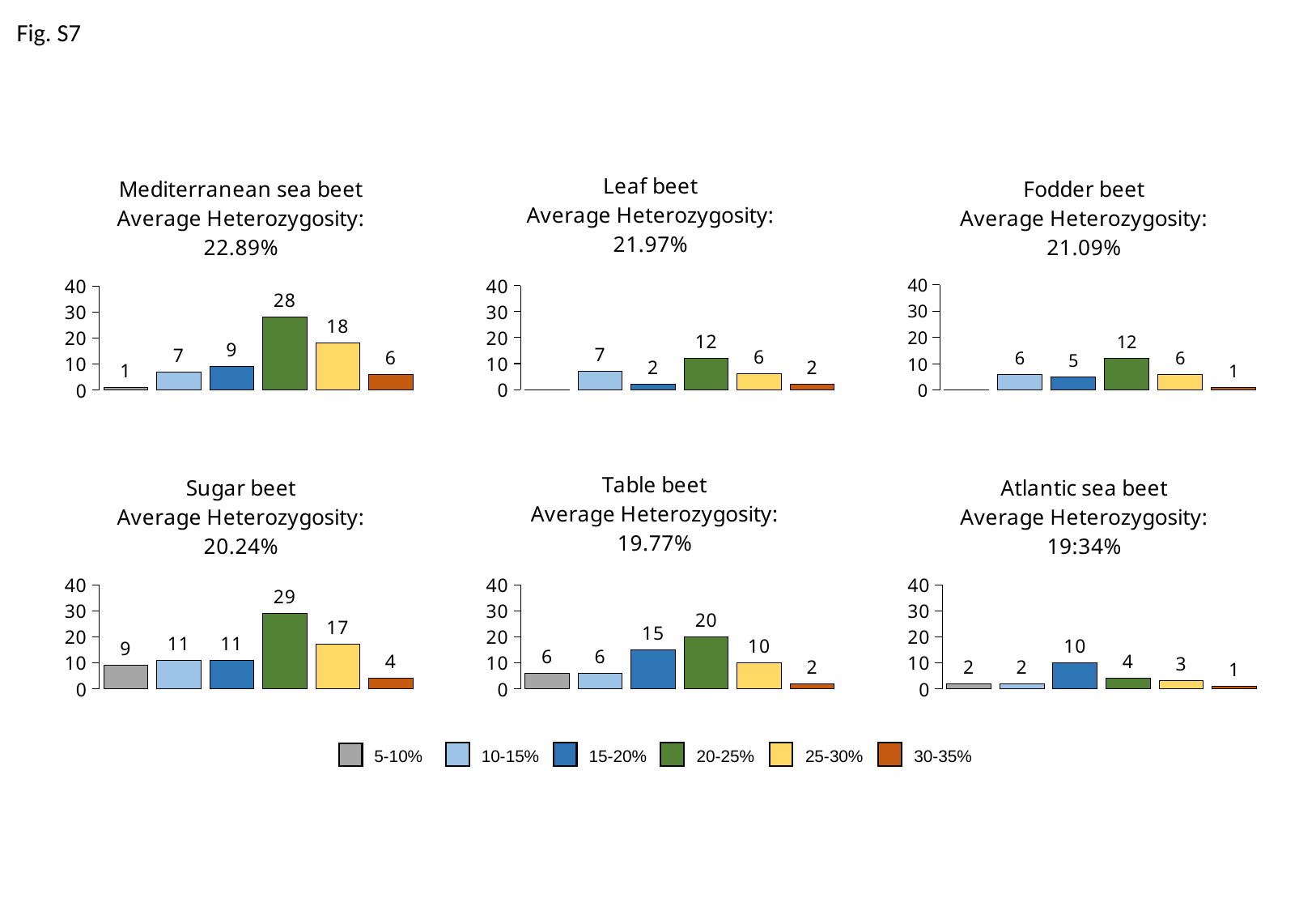

Fig. S7
### Chart: Leaf beet
Average Heterozygosity: 21.97%
| Category | |
|---|---|
| 5 | 0.0 |
| 10 | 7.0 |
| 15 | 2.0 |
| 20 | 12.0 |
| 25 | 6.0 |
| 30 | 2.0 |
### Chart: Fodder beet
Average Heterozygosity: 21.09%
| Category | |
|---|---|
| 5 | 0.0 |
| 10 | 6.0 |
| 15 | 5.0 |
| 20 | 12.0 |
| 25 | 6.0 |
| 30 | 1.0 |
### Chart: Mediterranean sea beet
Average Heterozygosity: 22.89%
| Category | |
|---|---|
| 5 | 1.0 |
| 10 | 7.0 |
| 15 | 9.0 |
| 20 | 28.0 |
| 25 | 18.0 |
| 30 | 6.0 |
### Chart: Sugar beet
Average Heterozygosity: 20.24%
| Category | |
|---|---|
| 5 | 9.0 |
| 10 | 11.0 |
| 15 | 11.0 |
| 20 | 29.0 |
| 25 | 17.0 |
| 30 | 4.0 |
### Chart: Table beet
Average Heterozygosity: 19.77%
| Category | |
|---|---|
| 5 | 6.0 |
| 10 | 6.0 |
| 15 | 15.0 |
| 20 | 20.0 |
| 25 | 10.0 |
| 30 | 2.0 |
### Chart: Atlantic sea beet
Average Heterozygosity: 19:34%
| Category | |
|---|---|
| 5 | 2.0 |
| 10 | 2.0 |
| 15 | 10.0 |
| 20 | 4.0 |
| 25 | 3.0 |
| 30 | 1.0 |25-30%
30-35%
5-10%
10-15%
15-20%
20-25%

## Slide 8
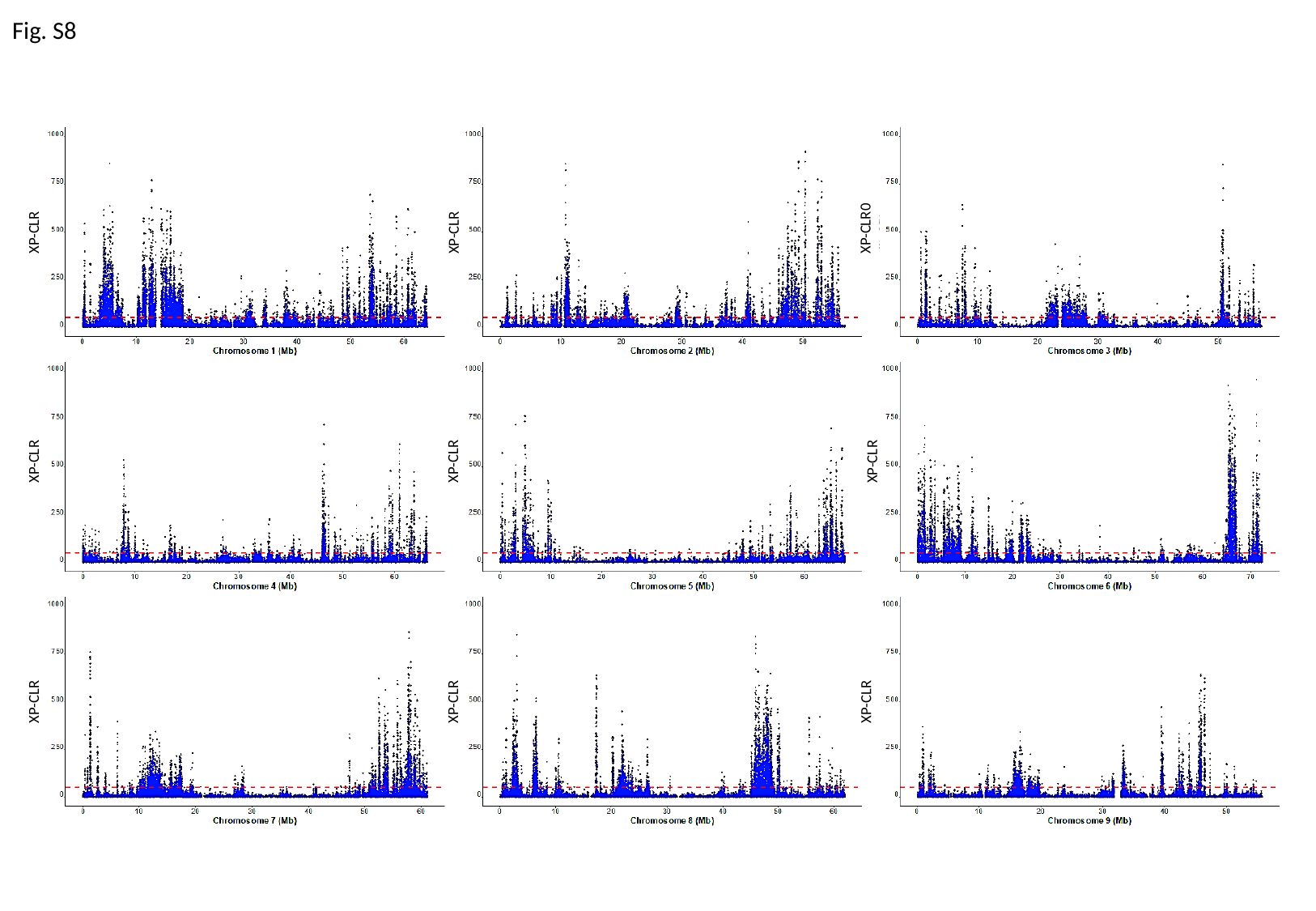

Fig. S8
XP-CLR
XP-CLR0
XP-CLR
XP-CLR
XP-CLR
XP-CLR
XP-CLR
XP-CLR
XP-CLR

## Slide 9
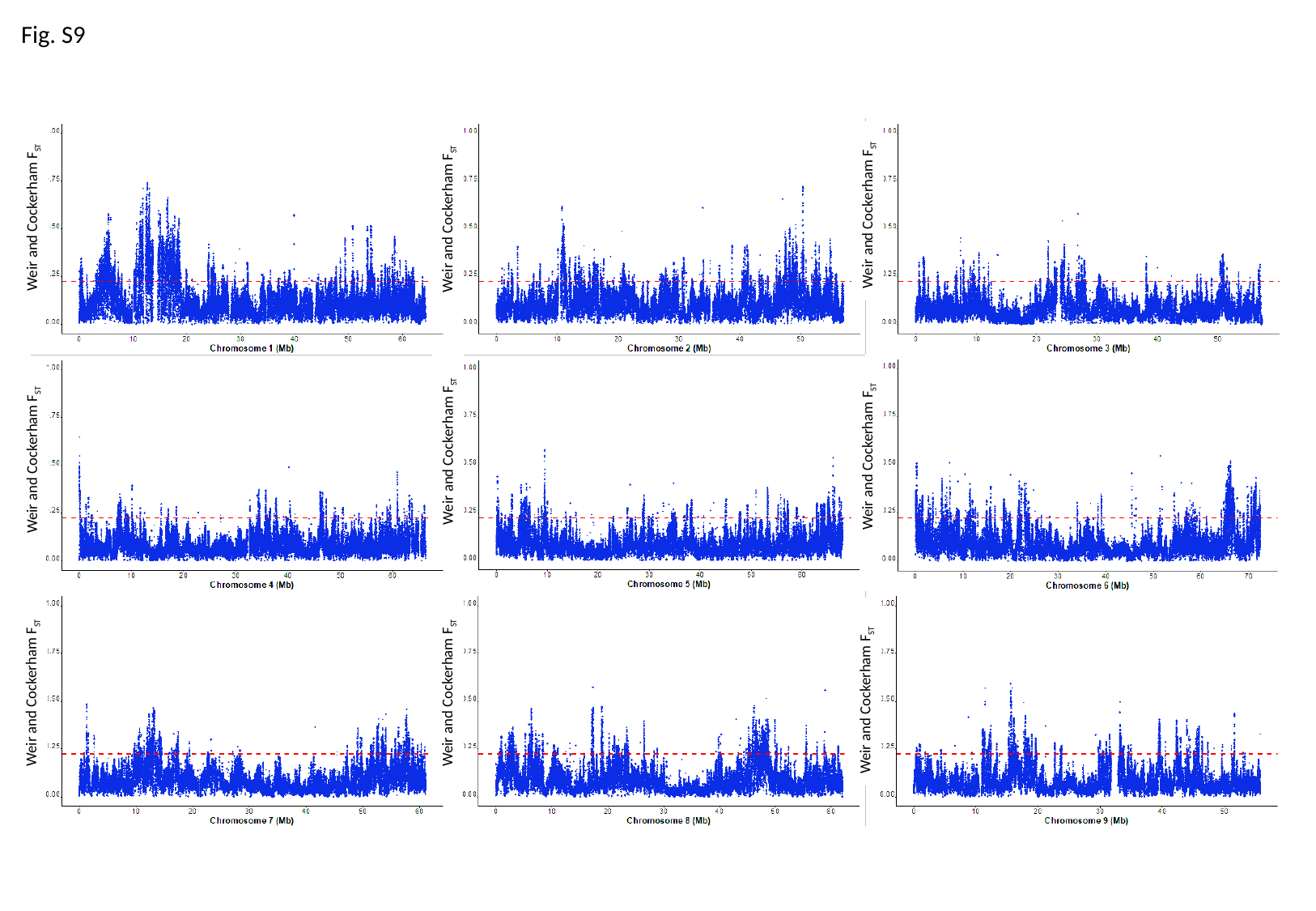

Fig. S9
Weir and Cockerham FST
Weir and Cockerham FST
Weir and Cockerham FST
Weir and Cockerham FST
Weir and Cockerham FST
Weir and Cockerham FST
Weir and Cockerham FST
Weir and Cockerham FST
Weir and Cockerham FST

## Slide 10
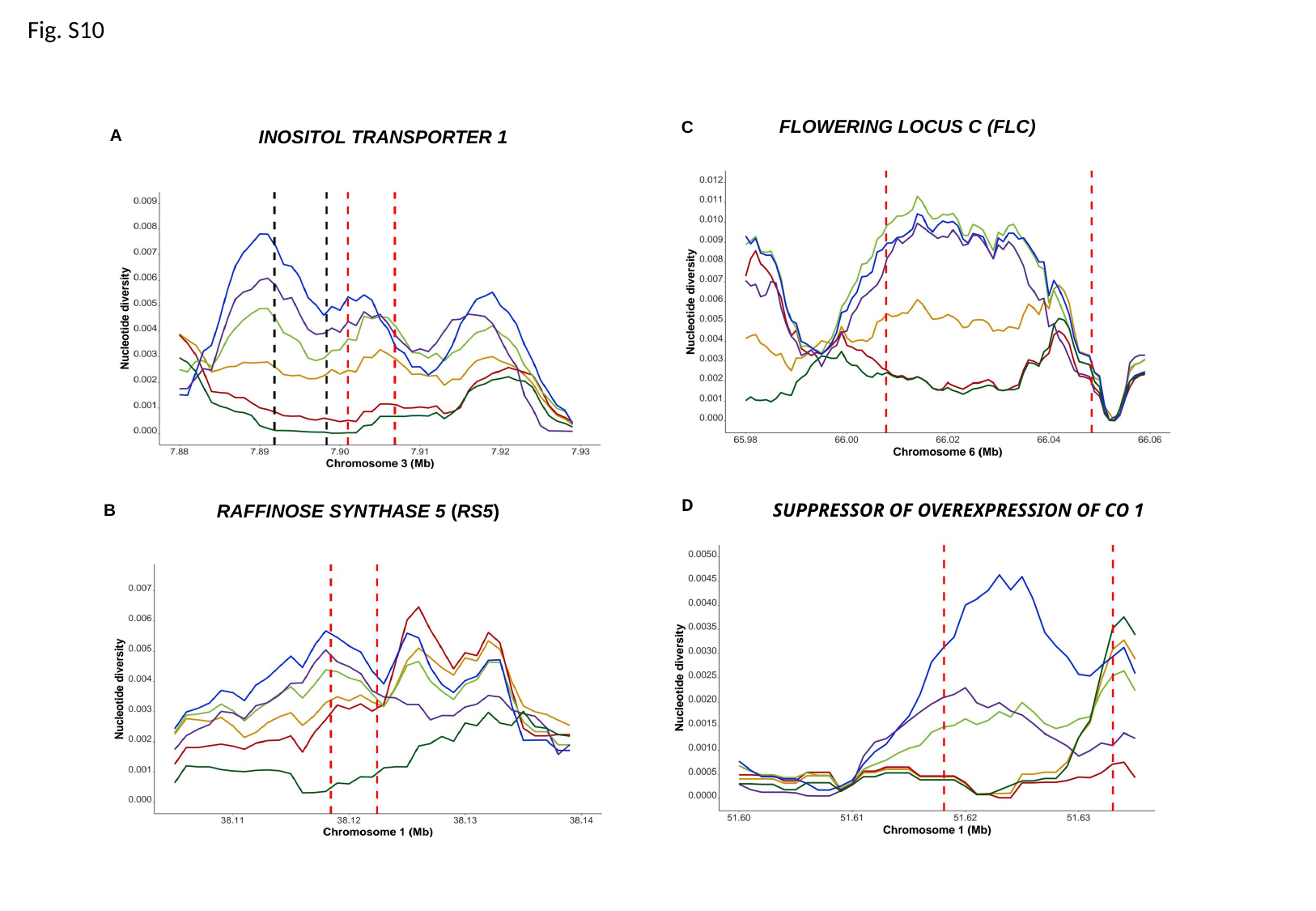

Fig. S10
Flowering locus C (FLC)
C
A
Inositol transporter 1
D
SUPPRESSOR OF OVEREXPRESSION OF CO 1
B
Raffinose Synthase 5 (RS5)

## Slide 11
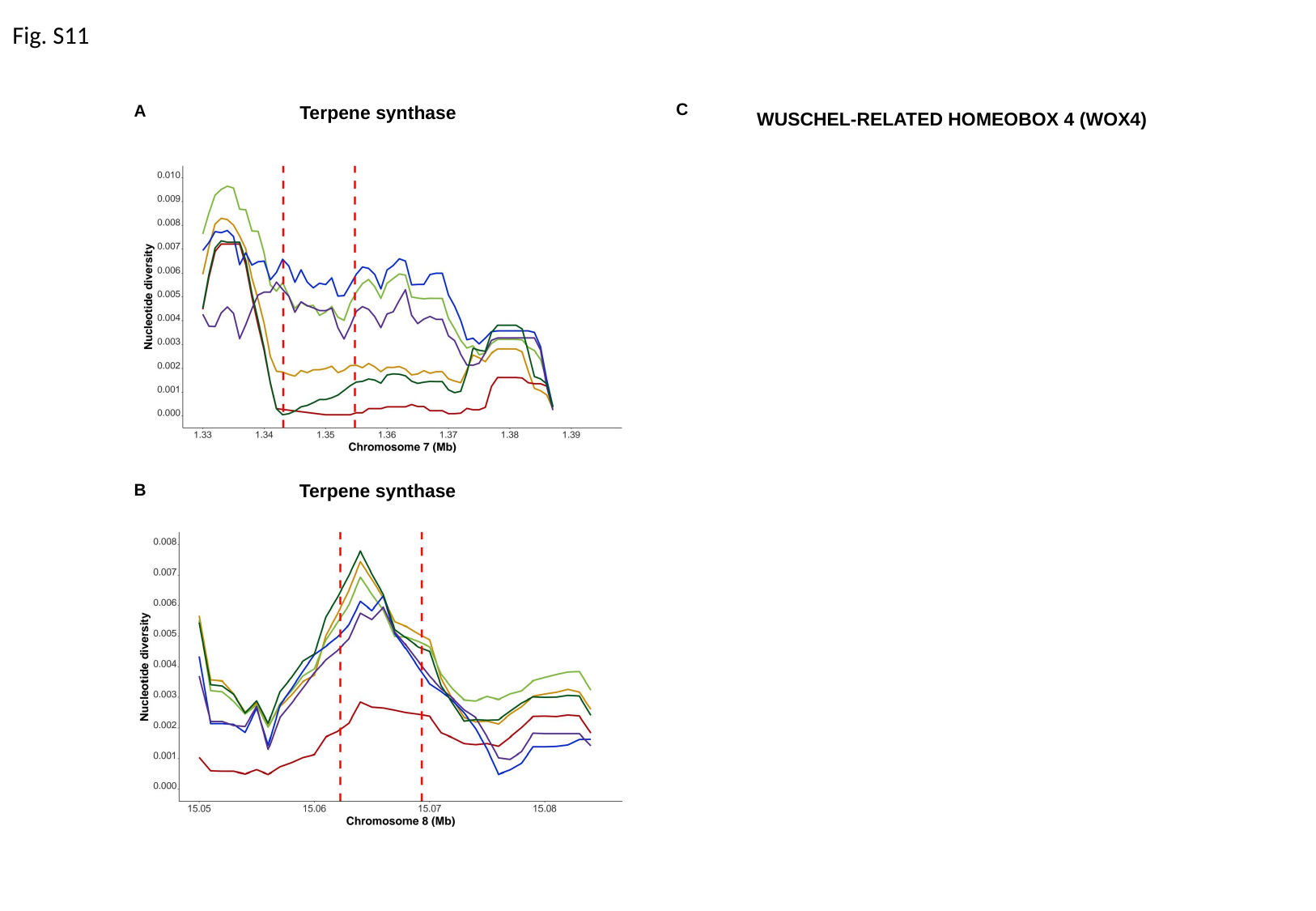

Fig. S11
C
A
Terpene synthase
WUSCHEL-RELATED HOMEOBOX 4 (WOX4)
B
Terpene synthase

## Slide 12
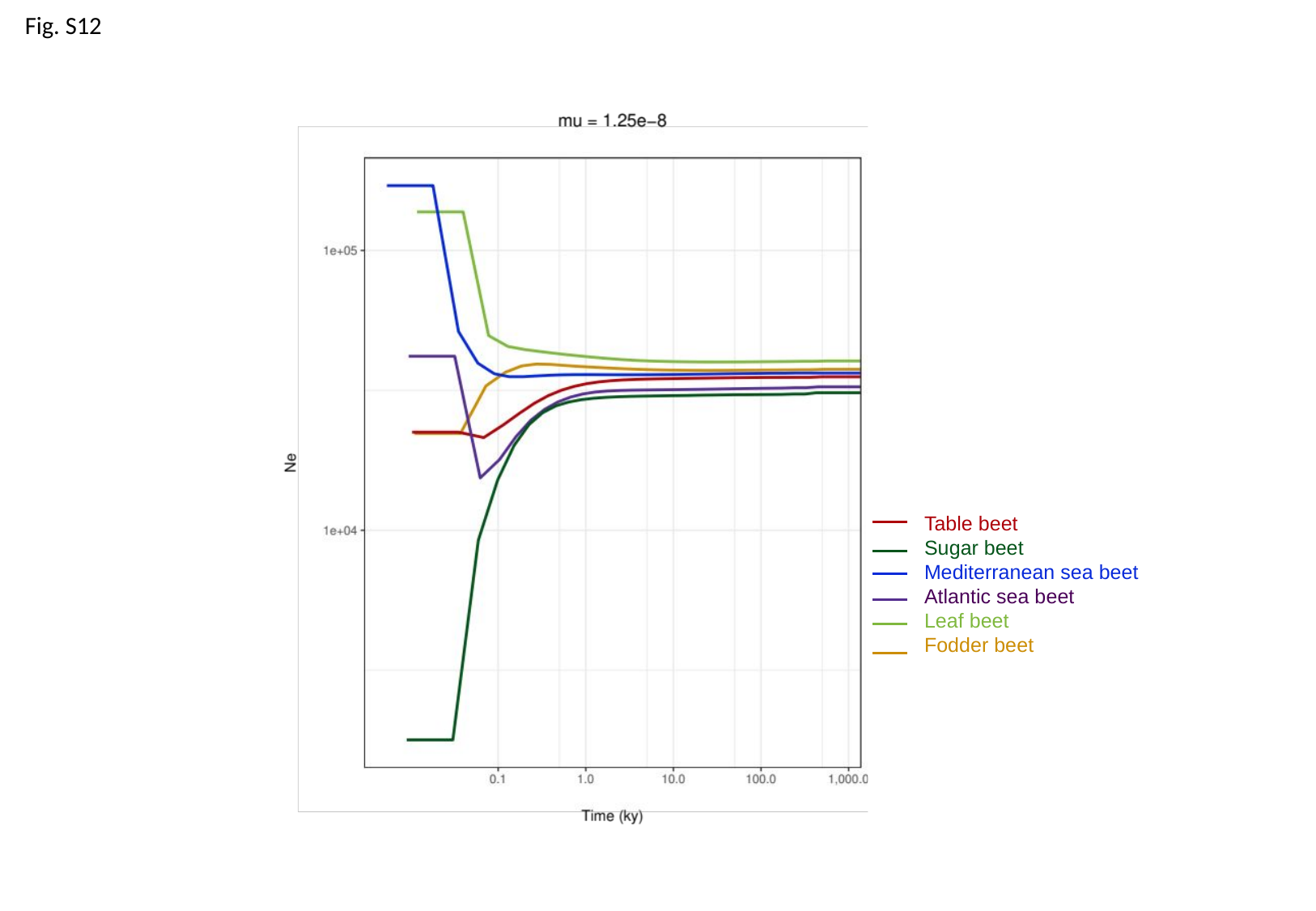

Fig. S12
Table beet
Sugar beet
Mediterranean sea beet
Atlantic sea beet
Leaf beet
Fodder beet

## Slide 13
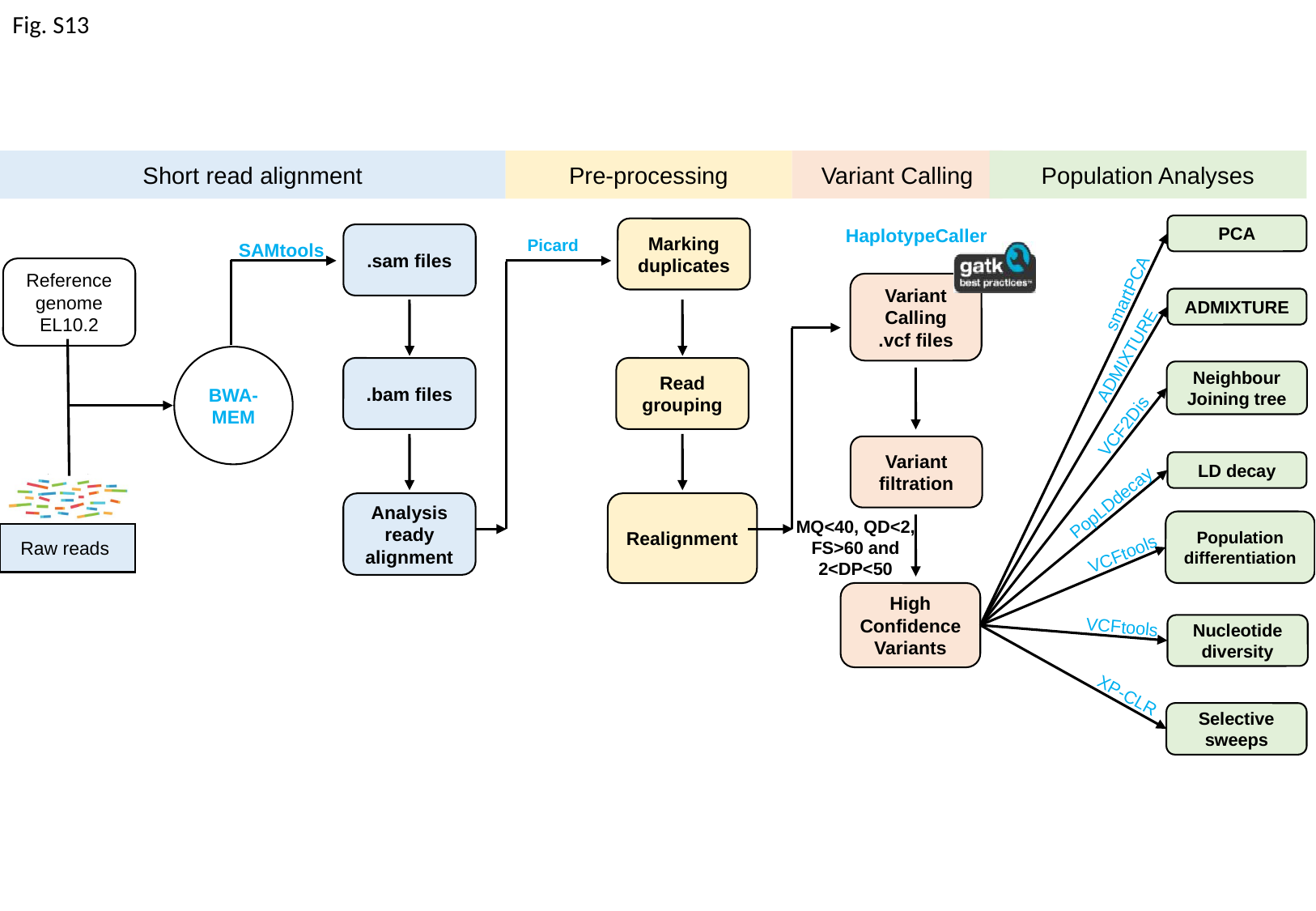

Fig. S13
Variant Calling
Short read alignment
Pre-processing
HaplotypeCaller
Marking duplicates
SAMtools
.sam files
Picard
Reference genome EL10.2
Variant Calling
.vcf files
BWA-MEM
.bam files
Read grouping
Analysis ready alignment
Realignment
Raw reads
High Confidence Variants
Population Analyses
PCA
smartPCA
ADMIXTURE
ADMIXTURE
Neighbour Joining tree
VCF2Dis
Variant filtration
LD decay
PopLDdecay
MQ<40, QD<2,
FS>60 and 2<DP<50
Population differentiation
VCFtools
VCFtools
Nucleotide diversity
XP-CLR
Selective sweeps
