# Supplementary material for: Signatures in domesticated beet genomes pointing at genes under selection in a sucrose-storing root crop
Source: BMC Biol. 2025 Oct 7;23:299. doi: 10.1186/s12915-025-02422-5 (PMC12505646; doi:10.1186/s12915-025-02422-5)
Supplement: Supplementary file 1 — Additional file 1: Table S1: Summary statistics of the paired-end Illumina raw reads without trimming from 290 accessions. Table S2: Summary statistics of the paired-end Illumina trimmed and cleaned reads from 290 accessions. Table S3: Summary statistics of the sample information, total mapped reads (%), sequencing depth and genome coverage (%) across 290 accessions. “NA” : Not Available. Table S4: Summary statistics of sequence variants (SNPs and INDELs) in 290 beet accessions compared to the EL10 reference genome. EL10.2_2 genome assembly comprises 568.8 Mb and is assembled into 18 pseudomolecules. In total, 564.2 Mb (99.2%) of the total genome assembly is organized into 9 main chromosomes (pseudomolecules), and the remaining 4.6 Mb of the genome is placed on 9 small unscaffolded pseudomolecules. Fig. S1: A bar plot illustrating the number of variants, including SNPs and INDELs, across all nine EL10 chromosomes in the Beta mini-core collection of 290 accessions. The Y-axis represents the total number of variants, and the X-axis corresponds to the nine EL10 chromosomes. Chromosome 9 exhibits the minimum number of variants, approximately 1.1 million, while Chromosome 5 displays the maximum number, around 1.5 million variants. On average, each chromosome harbors approximately 1.3 million variants. Table S5: Summary statistics of the number of SNPs and INDELs with their predicted effect using VEP across 290 accessions. Table S6: Summary statistics of the number of SNPs and INDELs within coding sequences with their predicted effect using VEP across 290 accessions. Fig. S2: Principal component analysis (PCA) of 290 beet accessions comparing second and third principal components. PC2 and PC3 represent the second and third components, accounting for 3.08% and 2.50% of the total variation, respectively. The colors represent different genetic clusters according to their beet type, with the following accessions: wild accession from the Atlantic (purple), Mediterranean (blue) [file 12915_2025_2422_MOESM1_ESM.zip › Supplementary Note 1.docx]

**Supplementary Note 1**

if (!requireNamespace("BiocManager", quietly=TRUE))

install.packages("BiocManager")

BiocManager::install("gdsfmt")

BiocManager::install("SNPRelate")

### install the development version of package from Github ###

### install_github requires that user build from source i.e. make command and compliers must be installed on your system ###

library("devtools")

# install_github("zhengxwen/gdsfmt")

install_github("zhengxwen/SNPRelate")

### loadding the package ####

library(gdsfmt)

library(SNPRelate)

library(ggplot2)

######## Genome_wide_PCA ##################

### Filtered SNP file using plink file with min DP 5, max missing 0.8, biallelic ###

bed.fn <- "MeanDP5_Missingness_0.9_Maf_0.05_Biallelic_Variant_only_Genotype_filter_Hard_filtered_SNPs_diversity_panel_default_without_boku_rename.bedformat.bed"

fam.fn <- "MeanDP5_Missingness_0.9_Maf_0.05_Biallelic_Variant_only_Genotype_filter_Hard_filtered_SNPs_diversity_panel_default_without_boku_rename.bedformat.fam"

bim.fn <- "MeanDP5_Missingness_0.9_Maf_0.05_Biallelic_Variant_only_Genotype_filter_Hard_filtered_SNPs_diversity_panel_default_without_boku_rename.bedformat.bim"

snpgdsBED2GDS(bed.fn, fam.fn, bim.fn, "beet_panel.gds", cvt.chr="char")

# of fragments: 18 ###integer/chromosome number###

beet_genofile<- snpgdsOpen("beet_panel.gds")

# Run PCA

pca_beet_and_wild <- snpgdsPCA(beet_genofile, num.thread=50, autosome.only = FALSE)

beet_and_wild_first_PCs <- data.frame(sample.id = pca_beet_and_wild$sample.id,

PC1=pca_beet_and_wild$eigenvect[,1], # the first eigenvector

PC2=pca_beet_and_wild$eigenvect[,2],

PC3=pca_beet_and_wild$eigenvect[,3],

PC4=pca_beet_and_wild$eigenvect[,4],

PC5=pca_beet_and_wild$eigenvect[,5],

PC6=pca_beet_and_wild$eigenvect[,6],

PC7=pca_beet_and_wild$eigenvect[,7],

PC8=pca_beet_and_wild$eigenvect[,8],

PC9=pca_beet_and_wild$eigenvect[,9],

PC10=pca_beet_and_wild$eigenvect[,10],

stringsAsFactors = FALSE)

beet_and_wild_first_PCs

write.csv(beet_and_wild_first_PCs,"beet_panel_10M_first_PCs.csv",quote = F,row.names = F)

# variance proportion (%)

pc.percent <- pca_beet_and_wild$varprop*100

pc.percent

write.csv(pc.percent,"pc.percent",quote = F,row.names = F)

head(round(pc.percent, 2))

eigenvalue <- pca_beet_and_wild$eigenval

write.csv(eigenvalue, "eigenvalues", quote = F,row.names = F)

#save.image()

beet_panel <- read.csv("beet_types.csv", header = TRUE)

beet_and_wild_first_PCs["CROP_TYPE"] <- beet_panel$CROP_TYPE

ggplot(beet_and_wild_first_PCs,aes(PC1,PC2, colour=CROP_TYPE))+

scale_x_continuous(name="PC1 (X %)")+

scale_y_continuous(name="PC2 (X %)")+

ggtitle("Principal component analysis (PCA) of the Beta mini core collection") +

geom_point(size=4)+

theme_classic()+

scale_color_manual(values = c("#cc8d04","#ad0309","#7fb83e","#072ad9", "#03541b")) +

theme(plot.title = element_text(size = 26, hjust = 0.3, vjust = 5, face = "bold",family="serif"),

axis.title.x = element_text(size=22, color = "black", hjust = .5, vjust = -5, face = "bold",family="serif"),

axis.title.y = element_text(size=22, color = "black", hjust = .5, vjust = 5, face = "bold",family="serif"),

axis.text.x = element_text(size = 18, color = "black", hjust = .5, vjust = 0.5, angle = 45, family="serif"),

axis.text.y = element_text(size = 18, color = "black", hjust = .5, vjust = 0.4, family="serif"),

legend.title = element_text(size = 18, family="serif"),

legend.text = element_text(size = 18, family="serif"),

plot.margin = unit(c(2,2,2,2),"cm"))

ggsave("PC5_vs_PC5.png", dpi=600, units="in", width=16, height=9)

######## Genome_wide_heterozygosity ##################

heterozygosity <- read.table("plink.het", header=TRUE)

heterozygosity$HOM_PERC <- (heterozygosity$O.HOM./heterozygosity$N.NM.)*100

heterozygosity$HET_PERC <- 100-heterozygosity$HOM_PERC

write.table(heterozygosity, file="Heterozygosity.tsv", sep = "\t",row.names = FALSE)

#### plotiing using ggplot2 #####

library(ggplot2)

heterozygosity <- read.table("Heterozygosity.tsv", header = TRUE)

legend_title <- "CROP TYPE"

ggplot(data=heterozygosity, aes(x=CROP_TYPE, y=HET_PERC, color=CROP_TYPE)) +

#geom_violin()+

geom_boxplot(width=0.3) +

#stat_summary(fun=mean, geom='point', shape=20, size=8) +

scale_y_continuous(breaks = seq(0,40,5), limits=c(5,35))+

geom_jitter(position=position_jitter(0.1))+

scale_color_manual(values=c("#cc8d04","#ad0309","#7fb83e","#072ad9", "#552f8f" ,"#03541b"))+

labs(y="Percentage of heterozygous SNP sites (%)", x="Crop type") +

theme_classic()+

theme(plot.margin = unit(c(3, 3, 3, 3), "cm"))+

theme(axis.text.x = element_text(vjust = -1.0)) +

theme(axis.title.x = element_text(vjust = -2.0)) +

#theme(axis.text.x = element_text(angle = 45)) +

theme(axis.text=element_text(size=16,colour = "black"), axis.title=element_text(size=20))+

theme(legend.text = element_text(size = 16))+

theme(axis.title.y = element_text(vjust = 3.5))

ggsave("Violin_plot_heterozygosity_5_35.png", dpi=600, units="in", width=16, height=9)

######## Genome_wide_Pi ##################

library(ggplot2)

library(dplyr)

pi <- read.table("Chr_1_9_all_cluster_10kbwindow_1kbstep.pi", header=TRUE)

pi$CHROM <- as.numeric(as.character(pi$CHROM))

ggplot(pi, aes(x=pi$CROP_TYPE, y=pi$PI, color=CROP_TYPE)) + xlab("Crop types") + ylab("Nucleotide diversity (Pi)") +

theme_classic()+

ggtitle("Nucleotide diversity (Pi) across different crop types") +

theme(plot.title = element_text(size = 36, hjust = 0.5, face = "bold"),

axis.title.x = element_text(size=18, color = "black", hjust = .5, vjust = -5, face = "bold"),

axis.title.y = element_text(size=18, color = "black", hjust = .5, vjust = 4, face = "bold"),

axis.text.x = element_text(size = 18, color = "black", hjust = .5, vjust = -1),

axis.text.y = element_text(size = 18, color = "black", hjust = .5, vjust = 0.4),

legend.title = element_text(size = 20),

legend.text = element_text(size = 20),

plot.margin = unit(c(1,1,1,1),"cm")) +

geom_boxplot(width=0.4, size=1,outlier.size=1) +

scale_color_manual(values = c("#cc8d04","#ad0309","#7fb83e","#072ad9","#552f8f", "#03541b")) +

stat_summary(fun.y=mean, geom="point", size=5, color="red")
